# Deep-learning of Nanopore sequences reveals the 6mA distribution and dynamics in human gut microbiome

**DOI:** 10.1093/nsr/nwaf120

**Published:** 2025-03-29

**Authors:** Jiabao Cao, Yuqing Zhang, Wenhui Zhang, Faming Zhang, Jun Wang

**Affiliations:** CAS Key Laboratory of Pathogenic Microbiology and Immunology, Institute of Microbiology, Chinese Academy of Sciences, China; CAS Key Laboratory of Pathogenic Microbiology and Immunology, Institute of Microbiology, Chinese Academy of Sciences, China; University of Chinese Academy of Sciences, China; CAS Key Laboratory of Pathogenic Microbiology and Immunology, Institute of Microbiology, Chinese Academy of Sciences, China; University of Chinese Academy of Sciences, China; Medical Center for Digestive Diseases, The Second Affiliated Hospital of Nanjing Medical University, China; Key Lab of Holistic Integrative Enterology, Nanjing Medical University, China; CAS Key Laboratory of Pathogenic Microbiology and Immunology, Institute of Microbiology, Chinese Academy of Sciences, China; University of Chinese Academy of Sciences, China

In contrast to eukaryotes, the N6-methyladenine modification, or 6mA, is an overwhelmingly dominant genomic DNA modification in bacteria and archaea [1]. Evidence suggests that 6mA plays a role in regulating bacterial chromosome replication and altering chromosome formation [2–4]. Furthermore, it is associated with heterogeneity in gene expression patterns within the same bacterial population, which can lead to clinically important phenotypic states such as the production of pathogen surface antigens and toxins [5–8]. The 6mA methylome has been mapped for more than 2000 cultured species of prokaryotes, predominantly bacteria [9], however, there is paucity of data available on the methylome of complex microbiomes, largely due to technical challenges (See Supplementary introduction).

To comprehensively profile the methylome of the microbiome, we developed a deep-learning-based tool, methCaller, which accurately predicts genomic 6mA methylation from Oxford Nanopore Technologies (ONT) reads. We then evaluated its generalization ability and performance (Figs S1–S3, Supplementary primary analysis results). Subsequently, methCaller was employed to determine the 6mA methylome in human gut microbiome data from a cohort of 100 healthy individuals (Cross-sectional cohort, Supplementary methods). Metagenomic Assembled Genomes (MAGs) were previously obtained from this cohort using hybrid sequencing and assemblies with ONT and Illumina reads. Of 690 MAGs, 168 achieved 10× depth of coverage and 80% breadth of coverage in terms of ONT reads, making them suitable for 6mA profiling. A total of 4 074 065 6mA sites were identified in the MAGs, with a range of 3088 to 65 478 per MAG and 2174 to 18 759 per Mb of genome. The MEME tool was employed to identify 29 non-degenerate motifs, of which 18 were already present in the REBASE database (as of 4 March 2024; see Table S1) and the remaining 11 had not been previously reported.

With respect to motif usage within individual bacterial taxa, a phylogenetic tree was constructed using the aforementioned 168 MAGs, and the modification ratios of various motifs in each genome were compared. Four 6mA sequence motifs are widely distributed and present in >5 bacterial species with moderate to high modification ratios in the genome, including the CAGCAG motif (22.4%–63.7%), CCATC (60%–92.1%), CATCC (21%–69.5%), and GAAGG (27%–94.8%). Furthermore, 4 additional motifs are uniquely distributed in individual bacterial species with high methylation ratios, including CAAATGC (85.5%) in the species *Agathobacter*, CCAATG (87.3%) in *Parabacteroides*, GAGAAC (82.4%) in *Prevotellamassilia*, and RAATTY (94.1%) in *Akkermansia muciniphila* (Fig. S4A). Our findings suggest that the utilization of bacterial motifs in our healthy cohort exhibits taxonomic specificity. Furthermore, irrespective of motif usage, 6mA methylation levels were relatively more stable within species than across different species. (Fig. S4B, one-way ANOVA, *P* = 1.26e-13).

The 6mA methylome has been reported to be highly dynamic [10] and we therefore set out to investigate the relative stability of the 6mA methylome in the gut microbiome of healthy individuals. To this end, 10 healthy individuals were monitored for 10 days (Time-series cohort, Supplementary methods). Despite the potential limitations on statistical power resulting from the study's design, the present study aims to provide novel insights into the stability of 6mA methylation. Our statistical framework, named ‘methDiff’ (see Methods), revealed significant inter-individual variation in 6mA methylation levels in six bacteria with >5 occurrences, as well as relatively stable levels within the same individual (Fig. 1A, Wilcoxon rank-sum test, one-sided *P* < 0.05). In particular, Principal Coordinate Analysis (PCoA) based on methDiff measures revealed that 6mA methylome of *Phocaeicola vulgatus, Bifidobacterium pseudocatenulatum, Bacteroides stercoris*, and *Bifidobacterium adolescentis* showed significant inter-individual variation (Fig. 1B, PERMANOVA, *P* < 0.05). Therefore, within the time frame of our study, the 6mA methylome of specific bacterial species remains stable within the same host individuals, allowing for the distinction between different individuals.

**Figure 1.**
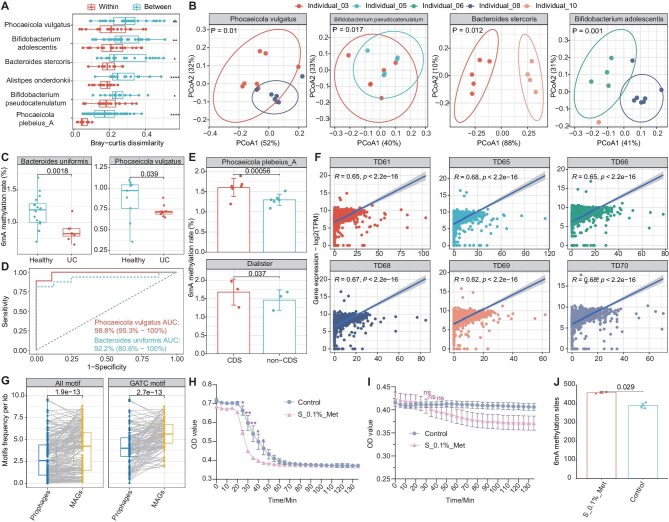
Analysis of the methylome of the human gut microbiome, including species-specific methylation patterns, differences between individuals, between health and disease, and functional implications. (A) Differences in bacterial genome methylation usage within and between individuals. The Bray–Curtis distance was calculated based on the 5-mer methylation usage of the bacterial genome. One-sided Wilcoxon rank sum test. *P < 0.05, **P < 0.01, ***P < 0.001, ****P < 0.0001. (B) Principal coordinates analysis (PCoA) ordination plot of 5-mer methylation profiles in bacterial genomes based on Bray–Curtis distances among different individuals. From left to right are the differences in 6mA methylation levels of *Phocaeicola vulgatus, Bifidobacterium pseudocatenulatum, Bacteroides stercoris*, and *Phocaeicola plebeius_A* between individuals. (C) Boxplot of bacterial 6mA methylation ratios between healthy individuals and UC patients. The left panel shows the methylation rate of *Bacteriodies uniformis*, and right panel *P. vulgatus*. One-sided Wilcoxon ran sum test. (D) ROC curves for random forest classification models. (E) 6mA methylation ratio of coding versus non-coding regions in the genomes of *P. plebeius_A* and *Dialister.* The *P*-values were obtained by paired t-test. (F) Scatterplot of the correlation between 6mA methylation density and gene expression of genes encoded by *P. plebeius_A* in the time-series cohort. (G) Boxplot of motif frequency in prophages and corresponding hosts. The number of motifs was corrected using genome length and expressed as the number of motifs per kb of genome length. The *P*-values were obtained using paired t-test. (H, I) Lysis curves of *Escherichia. coli* strains K12 and top10 infected by T1 phage. The light blue and pink curves represent the trends of OD values of *E. coli* in the M9 medium group and in the M9 medium supplemented with additional 0.1% methionine, respectively. Each set of experiments include 4 biological replicates, and error bars for each time point were plotted based on the mean and standard deviation. One-way ANOVA with Bonferroni's multiple comparison correction, *P < 0.05, **P < 0.01. (J) Number of 6mA methylation sites in the T1 phage genome, red: the number of T1 phage genome 6mA methylated after additional supplementation of 0.1% methionine in the medium, green: control group. Two-tailed Wilcoxon rank-sum test.

To gain insight into the underlying methylome differences between healthy individuals and those with ulcerative colitis (UC), we performed a comprehensive examination of the 6mA methylome in a further 100 patients using methCaller (UC cohort, Supplementary methods). The investigation focused on two MAGs (*Bacteroides uniformis* and *P. vulgatus*) that were identified in >5 cases in both the healthy and UC patient populations. First, it was observed that the methylation levels were significantly higher in healthy individuals than in UC patients (Fig. 1C, Wilcoxon rank-sum test, one-sided *P* < 0.05). Furthermore, the profiles of 6mA methylation, which were predominantly enriched in the coding genes of these two bacterial species, showed significant divergence between healthy individuals and UC patients (Fig. S5, PERMANOVA, both *P* < 0.01). Based on these findings, we constructed a random forest machine learning model that can be used to effectively discriminate between the bacterial species present in healthy individuals and patients, with a precision of 87.5% and 88.9%, and a ROC AUC of 92.2% and 98.8%, respectively (Fig. 1D). In conclusion, significant differences were identified in the methylomes of individual gut microbial species in UC, which have the potential to serve as markers for disease diagnosis in the future.

We next investigated whether the levels of 6mA methylation in individual bacteria and at the gut microbiome have an impact on gene transcription. An initial examination of the methylation levels in *Escherichia coli* K12 and Top10, revealed a markedly elevated 6mA methylation frequency in the coding region (CDS) relative to the intergenic region (non-CDS) (Fig. S6A, paired t-test *P* = 2.568e-05). The combination of transcriptome data from both strains revealed a significant positive correlation between transcript expression levels and the number of 6mA methylation sites within the coding region, even after correction for genome length (Fig. S6B, both *R* > 0.5, *P* < 2.2e-16). Subsequently, our investigation focused on *Phocaeicola plebeius_A* and *Dialister* in the Time-series cohort, which were detected at multiple time points and had sufficient coverage for 6mA analysis (Fig. S7). As with the findings in *E. coli*, 6mA sites were significantly enriched in the coding regions of both species (Fig. 1E, paired t-test *P* < 0.05). Furthermore, a significant positive correlation was also identified between the number of 6mA sites in the CDS and the normalized gene expression for each gene (Fig. 1F, *R* > 0.6, *P* < 2.2e-16; Fig. S8, *R* > 0.35, *P* < 2.2e-16). Collectively, these results suggest that the presence of 6mA methylation in the CDS region may facilitate gene transcription, although the underlying mechanism remains to be elucidated.

It is acknowledged that epigenetic modification of bacterial genomic DNA is necessary for the Restriction-Modification (R-M) system, which serves to defend against phage infection and other foreign DNA [11,12]. Consequently, an additional examination was conducted on the 6mA methylation profiles of prophages in the healthy cohort (Cross-sectional cohort and Time-series cohort). A total of 746 phages were identified from 396 MAGs, with a range of 1 to 8 phages per MAG (Fig. S9A). Furthermore, a total of 26 835 6mA methylation sites and 25 motifs were identified in 160 phages (Fig. S9B). Notably, the frequency of 6mA motifs showed a pronounced disparity between phages and their hosts. This was particularly evident in the case of motifs that were dominant in bacterial species, where motifs from phages related to that species were used significantly less frequently, such as GATC (Fig. 1G). Given that bacterial restriction enzymes are capable of recognizing and cleaving unmethylated motifs in phages, the apparent strategy of ‘motif depletion’ may be a means of avoiding recognition and a defense against host defense mechanisms.

Besides the usage of 6mA motifs, we further tested the effect of changing 6mA levels in modulating the phage-host interactions. The lysis curves of wild-type *E. coli* K12 and a restriction enzyme-deficient strain, Top10, were observed under culture conditions with or without methionine after infection with T1 phage. The amount of K12 was significantly reduced in cultures supplemented with an additional 0.1% methionine compared to the control (Fig. 1H). However, this reduction was not observed in the TOP10 (Fig. 1I). Since methionine can be converted to S-adenosylmethionine in bacteria, providing a methyl precursor for methyltransferase, the methylation level of T1 phage was investigated in detail. The results demonstrated that methionine supplementation led to a significant increase in the level of 6mA methylation in comparison to the control (Fig. 1J, Wilcoxon rank-sum test, *P* < 0.05). This indicates that the elevated level of genomic methylation in the phage overcame the restriction enzyme recognition and cleavage in K12, thereby enhancing the efficiency of infection and lysis.

N6-methyladenine modification represents the predominant form of DNA methylation in bacteria and plays a pivotal role in various biological processes. Nevertheless, the intricate methylation patterns observed in the gut microbiome have remained largely uninvestigated. The results of our study indicate that 6mA is a temporally stable and highly personalized microbiome signature. Moreover, the 6mA profiles of specific bacteria can be employed to distinguish between healthy individuals and patients with typical diseases, such as ulcerative colitis. It is therefore recommended that future studies should prioritize elucidating functional differences, particularly those related to dysbiosis and meta-epigenomes. In terms of functional impact, our findings suggest that gene coding regions are enriched in 6mA in both *E. coli* and human gut microbiomes, and that both are positively correlated with higher levels of gene expression. Furthermore, phages circumvent host restrictive defense by depleting host-preferred motifs. Conversely, increasing 6mA methylation through methionine supplementation has been observed to enhance phage-mediated bacterial cell lysis. It seems reasonable to suggest that increasing 6mA modification levels by supplementing methyl donors such as methionine in phage therapies targeting drug-resistant bacteria could enhance overall efficacy.

## Supplementary Material

nwaf120_Supplemental_File
