# Supplementary material for: Deep-learning of Nanopore sequences reveals the 6mA distribution and dynamics in human gut microbiome
Source: Natl Sci Rev. 2025 Mar 29;12(6):nwaf120. doi: 10.1093/nsr/nwaf120 (PMC12118455; doi:10.1093/nsr/nwaf120)
Supplement: nwaf120_Supplemental_File [file nwaf120_supplemental_file.docx]

Deep-learning of Nanopore sequences revealing the 6mA distribution and dynamics in human gut microbiome

[Introduction 2](#_Toc193467767)

[Primary analysis results 3](#_Toc193467768)

[Methods 5](#_Toc193467769)

[Supplementary figure and table 14](#_Toc193467770)

[Figure S1. Construction and evaluation of methCaller. 14](#_Toc193467771)

[Figure S2. Evaluation of generalisation capabilities of methCaller. 15](#_Toc193467772)

[Figure S3. Performance comparison of methCaller against mCaller and Tombo. 16](#_Toc193467773)

[Figure S4. Landscape of 6mA methylation in human gut microbiome. 16](#_Toc193467774)

[Figure S5. 6mA methylation of gut microbiota distinguishes healthy individuals from UC patients. 17](#_Toc193467775)

[Figure S6. 6mA methylation in *E. coli* are positively correlated with gene expression. 18](#_Toc193467776)

[Figure S7. Distribution of 6mA methylation sites in intestinal bacterial genomes. 18](#_Toc193467777)

[Figure S8. 6mA methylation in *Dialister* are positively correlated with gene expression 19](#_Toc193467778)

[Figure S9. 6mA methylation of prophages in the human gut microbiome 19](#_Toc193467779)

[Supplementary Table 1. Denovo motifs identified by methCaller in human gut microbiome. 20](#_Toc193467780)

# **Introduction**

Unlike 5mC that can be reliably identified by bisulfite sequencing with the Illumina platform, 6mA is traditionally detected with restriction enzyme-based approach, and the availability of enzymes severely limits the targetable sequences^1-3^. Recently, 6mA profiling is mainly carried out using single-molecule, real-time (SMRT) sequencing platform such as Pacbio, which can discern 6mA from unmethylated sites by distinguishing inter-pulse duration (IPD) of the DNA polymerase during sequencing by synthesis – an approach that depends on ‘subreads’ level information but discontinued in the most recent versions of sequencing chemistry^4-6^. Alternatively based on Oxford Nanopore (ONT) reads, multiple methods have also been developed to detect 6mA based on the difference in electronic current signals between methylated vs unmethylated sites^7-11^; limited by the training data used (mainly *E. coli* in which >90% methylated sites are within GATC), all available methods have high error rate in detecting 6mA occurring in other diverse motifs. A different approach that requires both naïve and methylation-free DNA (by whole genome amplification) can achieve *de novo* detection without the consideration of motifs, expanding this to larger scale of bacterial strains and metagenomic samples however remains difficult^12^.

As the fast-developing Nanopore platform are considerably more scalable for bacterial genome and complex microbiome studies, especially that ONT sequencing of human metagenomes is quickly increasing to reach population-level, achieving higher quality genome assemblies and enabled detection of mobile elements and structural variations^13-15^. Thus, straightforward and accurate profiling of 6mA on ONT platforms is highly desired for meta-epigenetic studies. To address these challenges and achieve accurate 6mA identification, we have developed a deep learning-based tool (methCaller) that overcomes the motif limitation and directly detects 6mA methylation based on ONT reads. Upon applying this tool to human gut microbiome data for a cohort comprising hundreds of individuals, we discovered highly diverse 6mA methylation motifs that can be used to differentiate bacterial taxonomical groups, distinguish between individuals and healthy individuals from ulcerative colitis (UC) patients. Additionally, we demonstrate that 6mA methylation levels in the gene coding region are positively correlated with gene expression, and in phages increasing 6mA methylation levels improves the efficiency of infecting bacterial hosts.

# **Primary analysis results**

With the aim of eventually building a model to identify N6-methyladenine modifications from Oxford Nanopore data, we first generated a synthetic DNA library, sequenced it using the Oxford Nanopore PromethION platform, and used this dataset for model training (Fig. S1A). Briefly, the library comprised of synthetic 11-mer DNA with NNNNNANNNNN sequences, where the A is either adenine or N6-methlyadenine (this length gives access to six 6-mer sequences – the length covered by a signal ‘event’ in ONT R9.4 chips -- containing the focal adenine/N6-methlyadenine). After ligation to adaptors, the sequencing dataset comprised a total of 6,230,004 sequences covering 616,517 pairs of 11-mers with both methylated and unmethylated sequences. This 11-mer dataset represents 58.8% of the total sequence diversity, of which 281,425 (45.6%) reached >10-fold coverage depth.

After base-calling of the ONT reads, we were able to retrieve the signal events for paired 11-mers with either adenine or N6-methlyadenine, and construct a BiLSTM (Bi-directional Long Short-Term Memory) model that can distinguish between methylated and unmethylated 11-mers (Fig. S1B). Using 80% of the data (randomly selected) as the training set, a 10% subset as the validation set, and the remainder as the test set, we achieved a precision of 89.3%, a recall of 95.7% recall and an area-under-the-curve (AUC) of 98.6%. It is noteworthy that the entirety of the model training and evaluation process was executed employing exclusively our synthetic library, as all publicly available data could not cover the theoretical diversity of 6mA motifs. We then constructed a 6mA reference dataset using 'gold standard' PacBio on two *E. coli* strains, K12 and Top10, and tested the performance of our model. In order to ensure comprehensive coverage of all loci, the *E. coli* ONT data was subjected to sequencing to a depth of coverage exceeding 100X, with a sequencing breadth of coverage of 100%. We compared the methylated adenine (A) positions determined by PacBio with the methylated adenine (A) positions predicted by our model to calculate the true positive rate (TPR) and the false positive rate (FPR), and found that our model (hereafter named methCaller) achieved 93.0% and 87.7% precision, 97.9% and 98.9% recall, and AUC of 99.5% and 99.6%, respectively (Fig. S1C).

In addition, we found that despite the incomplete coverage in terms of total 11-mer diversity in the training dataset, i.e. while we only obtained sequencing data for 58.8% of the 11-mers out of all possible sequences (1,048,576), our deep learning model nonetheless has the capacity of distinguishing methylated vs. naïve 11-mers that were not yet sequenced, which can be termed as ‘generalization’. We investigated the generalization property of methCaller by several benchmarks: firstly, using a subset of 67.6% of paired 11-mers to train methCaller, we were able to distinguish methylated and naïve sites in the 32.4% of 11-mers that were omitted as test datasets (89.6% precision, 92.7% recall, AUC of 98.2%, Fig. S2A); secondly, in the aforementioned *E. coli* K12 and Top10 datasets, a total of 965 6mA modified 11-mers (864 in K12 and 302 in Top10) were not present in the methCaller training set, yet our approach identified these 6mA sites with 100% and 99.8% precision, 77.2% and 53.3% recall, and AUC of 93.4% and 90.2%, respectively (Fig. S2B). In short, we have developed a tool based on deep learning of ONT data and achieved high accuracy on highly diverse 6mA methylation motifs.

We then compared the performance of methCaller with that of mCaller and Tombo for 6mA identification, two tools available for direct 6mA methylation detection, but trained with only on *E coli* data and thus limited to reliable detection of only the GATC methylation motif, Of these, mCaller used four classifiers (neural network, random forest, naïve Bayes and logistic regression) to predict 6mA, while tombo adopt the Mann-Whitney U test to detect controlled DNA methylation in *E. coli.* For the *E. coli* K12 and Top10 datasets mentioned above, methCaller had the highest precision and recall, followed by mCaller and then Tombo (Fig. S3). Notably, when quantifying the accuracy of the model using receiver operating characteristic (ROC) curves and the area under the curve (AUC) of precision-recall curves (PR), methCaller achieved ROC AUC of 99.6% and 98.1% and a PR AUC of 98.8% and 76.6%, respectively. In contrast, only mCaller had comparable ROC AUC to methCaller (98.2% and 95.5%) but with lower PR AUC (70.6% and 45.6%), and Tombo was lower in all measures (ROC AUC of 83.9% and 81.6%; PR AUC of 7.3% and 7.3%) (Fig. S1D-E). A comparison was also made between methCaller and modPhred, a methylation integrated tool that relies heavily on the Guppy software (Version≥3.4). The result of this comparison indicates that methCaller is comparable to modPhred in terms of precision, but has a higher recall (methCaller 97.9% and 98.9% vs. modPhred 88.8% and 93%, for K12 and Top10 respectively). Thus, by using more diverse 6mA motifs in training, our methCaller is able to outperform current tools that predict 6mA methylation from ONT data.

# **Methods**

**Construction of deep learning model for 6mA methylation prediction**

Aiming to eventually build a model to identify N6-methyladenine modifications from Oxford Nanopore data, we initially generated a synthetic DNA library, sequenced it using the Oxford Nanopore PromethION platform, and used this dataset for model training. Briefly, the library comprised synthetic 11-mer DNA with NNNNNANNNNN sequences, where the A is either adenine or N6-methlyadenine (this length gives access to six 6-mer sequences – length covered by one signal ‘event’ in ONT R9.4 chips -- containing the focal adenine/N6-methlyadenine). After ligating to adaptors, we sequenced such synthetic libraries using ONT, resulting in the generation of 6mA-modified and -unmodified DNA signals containing diverse motifs. Library preparation and sequencing of 6mA-modified and -unmodified DNA was then performed on R9.4.1 flow cells using PromethION. Briefly, DNA libraries were prepared according to the manufacturer’s instructions of the Native Barcoding Kit (ONT, EXP-NBD104) and the Genomic DNA Ligation Kit (ONT, SQK-LSK109), whereas raw signal data acquisition, real-time base calling, and de-multiplexing were performed using the MinKNOW software (version 22.10.7) and Guppy (version 6.3.9) developed by ONT. In the final, the sequencing dataset comprised a total of 6,230,004 sequences covering 616,517 pairs of 11-mers with both methylated and unmethylated sequences. This 11-mer dataset represents 58.8% of the total sequence diversities, and among those 281,425 (45.6%) reached >10X coverage depth.

**Feature extraction and feature engineering**

The output of the synthetic libraries on ONT sequencing platform include signal features and sequence features. The signal features are six consecutive events generated by the 11-mer sequences we designed, each represented by the mean, standard deviation, and dwelling time. These events were extracted and mapped to corresponding positions on the reference sequences using the minmap2^16^ software (version 2.10-r761) and nanopolish^17^ eventalign function (version 0.14.0). Prior to training the model, the signal features such as mean, standard deviation and dwelling time of a given DNA fragment (i.e. 11-mer sequences) were normalized by pandas. Sequence features are one-hot encoded vector combinations, where each base is represented by a 4-element vector in ACTG order, where 1 represents the current nucleotide type and the rest are 0. For example, for a given sequence of a 6-mer event, the feature vectors are combinations of 4*6 vectors. Signal features and sequence features are arranged and concatenated in the order in which the events occur, and finally, for a sequence of the given 11-mer, we obtained a 6*27 dimensional feature vector as input to the model.

**Model architecture of methCaller**

In the process of sequencing with ONT, bases with modifications changes the electrical signals that are reflected in multiple, consecutive events. We accordingly developed a 6mA methylation prediction model using RNN neural network algorithms in multiple motifs. The central feature of our model is the introduction of an attention mechanism that allows the model to pay more attention to the important parts when processing input sequences (bi-directional, long-short term memory, or biLSTM). This structure is developed within the framework of Natural Language Processing and has been applied to process sequence data. The detailed architecture consists of (i) a linear layer (input_dim=27, out_dim=64), which transforms the input through the linear layer into the input dimensions of the LSTM layer (ii) a bidirectional LSTM layer (input_dim=64, hidden_dim=128, num_layer=3, bidirectional=True, batch_first=True), the output sequence is obtained through the bidirectional LSTM layer ;(iii) Attention layer, the output of the bi-directional LSTM is linearly transformed, and then the softmax activation function is applied to obtain the attention weights; (iv) Fully connected layer, the weighted LSTM outputs are mapped to the final output space through the fully connected layer. Additionally, there is a dropout layer before the fully connected layer and the dropout is set to 0.5. The model was optimized using the ADAM optimiser with a loss function of Cross entropy. The model was established with pyTorch (version 2.0.0, https://pytorch.org/) and available at https://github.com/caojiabao/methCaller.

**Evaluation metrics of performance**

Accuracy, precision, recall and F1-score were used as metrics to evaluate the performance of the model. The specific formulas are as follows:

$\text{accuracy = }\frac{\text{TP}\text{+TN}}{\text{TP+FP+TN+FN}}$ (1)

$\text{precision}\text{ }\text{=} \frac{\text{TP}}{\text{T}\text{P}\text{+FP}}$ (2)

$\text{recall}\text{ }\text{= }\frac{\text{TP}}{\text{M}}$ (3)

$\text{F1-score = }\frac{\text{2*precision*recall}}{\text{precision+recall}}$ (4)

In the above equation, TP and FP represent the number of true positives and false positives predicted by the model, TN and FN represent the number of true negatives and false negatives predicted by the model, and M represents the number of true positives in the dataset. Thus, accuracy is the percentage of correct predictions for all modified/unmodified bases in the dataset, precision is the percentage of correct predictions for modified bases, and recall is the number of correct predictions for modified bases divided by the number of true modified bases in the dataset. The F1 score is a trade-off between precision and recall.

**Model training**

The biLSTM model methCaller was trained with the above-mentioned sequence and signal features. To balance the training dataset, the 6mA-modified and -unmodified DNA data were filtered according to the 11-mer sequences, thus each sequence had a corresponding modified (1) and unmodified signal (0). To select appropriate hyperparameters, the dataset was partitioned according to an 8:1:1 ratio to construct training dataset, testing dataset and validation dataset. In addition, to avoid overfitting, an ‘early stop’ strategy was used to train the model, stopping model training and saving the model when its performance started to degrade. For hyperparameter tuning, we alternated hidden_dim to 64, 128 and 256, learning_rate to 0.001 and 0.0001, the number of layers of LSTM to 2 and 3, and batch_size to 64, 128 and 256 respectively.

**Model testing**

ONT data from wild-type *E. coli* k12 and Top10 strains were also used for evaluation of methCaller and other methods. Briefly, the fast5 and fastq data were mapped to the reference genome (*E. coli* K12 substrand MG1655) using the minimap2, and nanopolish to obtain the event alignment results. The methCaller (and other tools) then examined the features and predicts the methylation probability for the positions of interest. Finally, the 6mA profiles of the above two strains by Pacbio were used as a reference so as to assess the performance of the model. We used the threshold of coverage ≥ 20X and fraction ≥ 0.5 in Pacbio identification as positive 6mA methylation.

**ONT and pacbio sequencing data on *Escherichia coli* K12 and Top10**

DNA from wild-type *E. coli* strain k12 as well as restriction system-knockout Top10 was extracted and then sequenced on an R9.4.1 flow cell using a PromethION sequencer. Library preparation was performed according to the manufacturer’s instructions using the Native Barcoding Kit (ONT, EXP-NBD104) and the Genomic DNA Ligation Kit (ONT, SQK-LSK109). Raw data acquisition and real-time base calling were performed using the MinKNOW and Guppy. To obtain the 6mA DNA methylation references, DNA was also sequenced using the Pacbio RSII system and 6mA methylation was identified using to the SMRT Link tool (version 6.0.0).

**cDNA sequencing on** ***Escherichia coli* K12 and Top10 using ONT**

The RNA was extracted from *E. coli* K12 and TOP10 strains that had been cultured to logarithmic and plateau phases, respectively. This was achieved by using the QIAamp RNA Blood Mini Kit (Qiagen), in accordance with the instructions provided by the manufacturer. The RNA was then eluted with RNase-free water. The RNA was reverse transcribed into cDNA in the following process: First-strand cDNA was synthesised in a 20 µL reaction mixture containing 13 µL of purified RNA per sample and 100 pmol of primer Rrm (5'-GACCATCTAGCGACCTCCAC-NNNNN-3'). The synthesis of double-stranded cDNA was then initiated by the addition of 100 pmol of primer Rrm and Klenow fragments (3.5 U µL-1; Takara). Preparation of the cDNA library was undertaken accordance with the manufacturer's guidelines, employing the Native Barcoding Kit (ONT, EXP-NBD104) and the Genomic DNA Ligation Kit (ONT, SQK-LSK109). The library was then subjected to sequencing using the PromethION system. ONT MinKNOW was utilised for the acquisition of raw sequencing data, while Guppy was employed for the real-time base calling of the raw data. Subsequent to this, barcodes and adapter sequences were removed from the raw data using the qcat software (version 1.1.0) with default parameters. The calculation of gene expression of E. coli was performed using salmon (version 1.4.0) with the following command: ‘salmon quant --noLengthCorrection -p 30 -l U’.

**Comparison between methCaller and other available tools**

To compare the performance among tools, we also analyzed the *E. coli* k12 and Top10 ONT results with the mCaller (https://github.com/al-mcintyre/mCaller) and Tombo (https://github.com/nanoporetech/tombo). For mCaller, event alignment results were computed using nanopolish and minimap2, followed by methylation prediction using the mCaller.py script and the pre-trained model r94_model_NN_6_m6A.pkl. For Tombo, the raw reads were resquiggled to the reference using tombo-resquiggle, and then 6mA detection was performed using tombo detect_modifications alternative_model.

**Methylome analysis in human gut microbiome**

Gut microbiome samples from 100 healthy individuals (cross-sectional cohort) and 10-individual, 10 time-point samples (time-series cohort) has been previously sequenced by ONT and published; an additional 100 treatment-naïve ulcerative colitis (UC) patients, with age and gender matching to that of cross-sectional cohort was also included and gut microbiome sequenced by ONT (UC cohort). Sequencing and metagenome assembly has been described previously (see ref^15^).

The 6mA methylation of MAGs were predicted using methCaller. Briefly, each long read sample was first mapped to MAGs sequence using minimap2; MAGs with a depth of genome coverage greater than or equal to 10X and with a breadth of genome coverage greater than or equal to 80% were selected for subsequent analysis. Sequencing events were then extracted via the eventalign function of Nanopolish, and features were then extracted and computed using our script PrepareData.py. Finally, the probability of 6mA methylation per adenine in the reads was then predicted using the script MethCalling.py, and the fraction of possible methylation of each adenine in the genome was counted using the script MethPosSum.py. Methylation sites per 2kb genome was calculated using the seqkit^18^ sliding (version 2.2.0) and bedtools^19^ intersect (version 2.26.0) programs, and CDS gene regions predicted by Prokka^20^.

After obtaining methylation information for each sample, for each MAGs, we merged all samples with >10 X coverage and extracted characteristic sequences around each methylated adenine adjacent to 10 bases on the left and 11 bases on the right, which were used to identify novel motifs. To obtain non-degenerate and accurate motifs, the motifs of the above 22 nt sequences were characterized using MEME^21^ (version 5.5.4) by setting the lengths sequentially to 4-14 and adjusting the parameters to ‘-mod zoops -evt 1e-30’ and the final results were manually curated. The previously curated MTase-recognizing motifs were from the Restriction Enzyme Database (REBASE; http://rebase.neb.com/cgi-bin/msublist; As of March 4, 2024) for our comparison.

The ratio of methylated sites per genome in each sample was defined as the ratio of the number of methylated adenines to the total number of adenines. Similarly, the methylation ratio of each motif in each genome in each sample was determined by dividing the number of methylated motifs by the total number of motifs. To compare intestinal bacterial 6mA methylation levels between and within individuals in a time-series cohort, we developed an additional statistical framework based on the Barry-Curtis distance (termed methDiff). Counting the frequency of adenine-centered 5-mer DNA sequences in the bacterial genome, we then examined the number of 6mA methylated 5-mer sequences predicted by methCaller’s to obtain the ratio of 5-mer methylation, then used for calculating the Bray-Curtis distance between samples or MAGs.

**Identification of prophage from MAGs**

Prophages of MAGs in human gut microbiome were identified using VIBRANT^22^ (version 1.2.1) with default parameters, and only MAGs satisfying a length of more than 1000 nt and at least 4 ORFs per contig were used. A homology search for prophage was performed using the Diamond BLASTX algorithm with the Refseq209 virus database as a reference. The output was then analyzed by MEGAN^23^ (Community Edition Version 6.21.10) using the Least Common Ancestor (LCA) method to obtain taxonomic information.

**Meta-transcriptome analysis of time series samples**

RNA was extracted from ten consecutive days’ samples from a single individual in time-series cohort using the QIAamp RNA Blood Mini Kit (Qiagen) following the manufacturer’s instructions and eluted with RNase-free water. RNA was reverse transcribed to cDNA by reference to the previous method^35^, and first-strand cDNA was synthesized in a 20 µL reaction mixture containing 13 µL of purified RNA per sample and 100 pmol of primer Rrm (5’-GACCATCTAGCGACCTCCAC-NNNNN-3’). Double-stranded cDNA was synthesized by adding 100 pmol of primer Rrm and Klenow fragments (3.5 U µL-1; Takara). Random amplification was performed with an 8 µL double-stranded cDNA template in a final reaction volume of 200 µL containing 4 × 10^-6^ M primer Rrm (5’-GCCGGAGCTCTGCAGAATTC-3’), 90 × 10^-6^ M dNTPs, 80 × 10^-6^ M Mg^2+^, 10X buffer, and 1 U KOD-Plus DNA polymerase (KOD-201). PromethION cDNA library preparation was performed according to the manufacturer’s instructions for native barcoding kit (ONT, EXP‐NBD114) and genomic DNA ligation kit (ONT, SQK‐LSK109). ONT MinKNOW was used to collect raw sequencing data, and Guppy was used for real‐time base calling of the raw data. Barcodes and adapter sequences were removed from the raw data using the qcat software (version 1.1.0) with default parameters. Post-qc long reads were mapped to single MAG reference genes using minimap2. The count of reads mapped to genes as well as gene expression was calculated using salmon (version 1.4.0) with the cmd ‘salmon quant --noLengthCorrection -p 30 -l U’.

**T1 phage lysis experiment**

*E. coli* K12 and Top10 strains were first cultured in LB and incubated until stabilization. Each *E. coli* strain was pre-cultured by transferring to M9 medium or M9 with additional 0.1% methionine, at 1% inoculum ratio at 37˚C, 220 rpm, for 16 h. The lysis curves were detected with a 96-well plate and a microplate reader (Cerillo, Stratus), in which 150 μl of bacteria and 4.5 μl of T1 phage were added to each well, and four replicate wells were set up for each group. The microplate reader was set to 37˚C and shaken at low speed for 20 seconds at 10 second intervals. OD600 was measured every 5 minutes for 24 times. Additional sampling was done at 30min and DNA was extracted using the QIAamp MinElute Virus Spin Kit (QIAGEN), and then sequenced as bacterial DNA on ONT PromethION.

**Statistics**

All statistical analysis was done in Rstudio using the R language (version 4.2.0). The model performance metrics were calculated using the functions of the R package ‘Metrics’, and the ROC and PR curves were computed and plotted using ‘pROC’ and ‘ggplot2’. Comparisons between two groups were made using the one sided or two sided Mann-Whitney-Wilcoxon rank sum test. Pairwise comparisons were performed using the paired t-test. Phylogenetic tree and heatmap of methylation motifs ratio were drawn using the R package ‘ggtree’. Multiple comparisons were performed using ordinary one-way analysis of variance (ANOVA) followed by Tukey’s multiple comparison test. Principal coordinate analysis (PCoA) was performed based on the 5-mer methylation profiles of MAGs across samples with the Bray–Curtis distance with R package ‘vegan’ and visualized with ‘ggplot2’, and multivariate analysis was performed using PER-MANOVA in vegan. Using the R package ‘randomForest’ and ‘caret’, a random forest classifier was constructed based on the 6mA usage in the CDS genes, and the ‘Leave-Group-Out Cross Validation’ (LGOCV) method was used for model training and test dataset resampling. Circos plot of methylation density in a single MAG genome was plotted using the R package ‘circlize’. The spearman correlation between the 6mA methylation level in CDS and gene expression was done with the stat_cor function in ‘ggpubr’ package. A sankey diagram of phage-host interactions was drawn using the R package ‘ggsankey’. The lysis curves were plotted using GraphPad Prism (version 10.2.2).

Supplementary figure and table

**
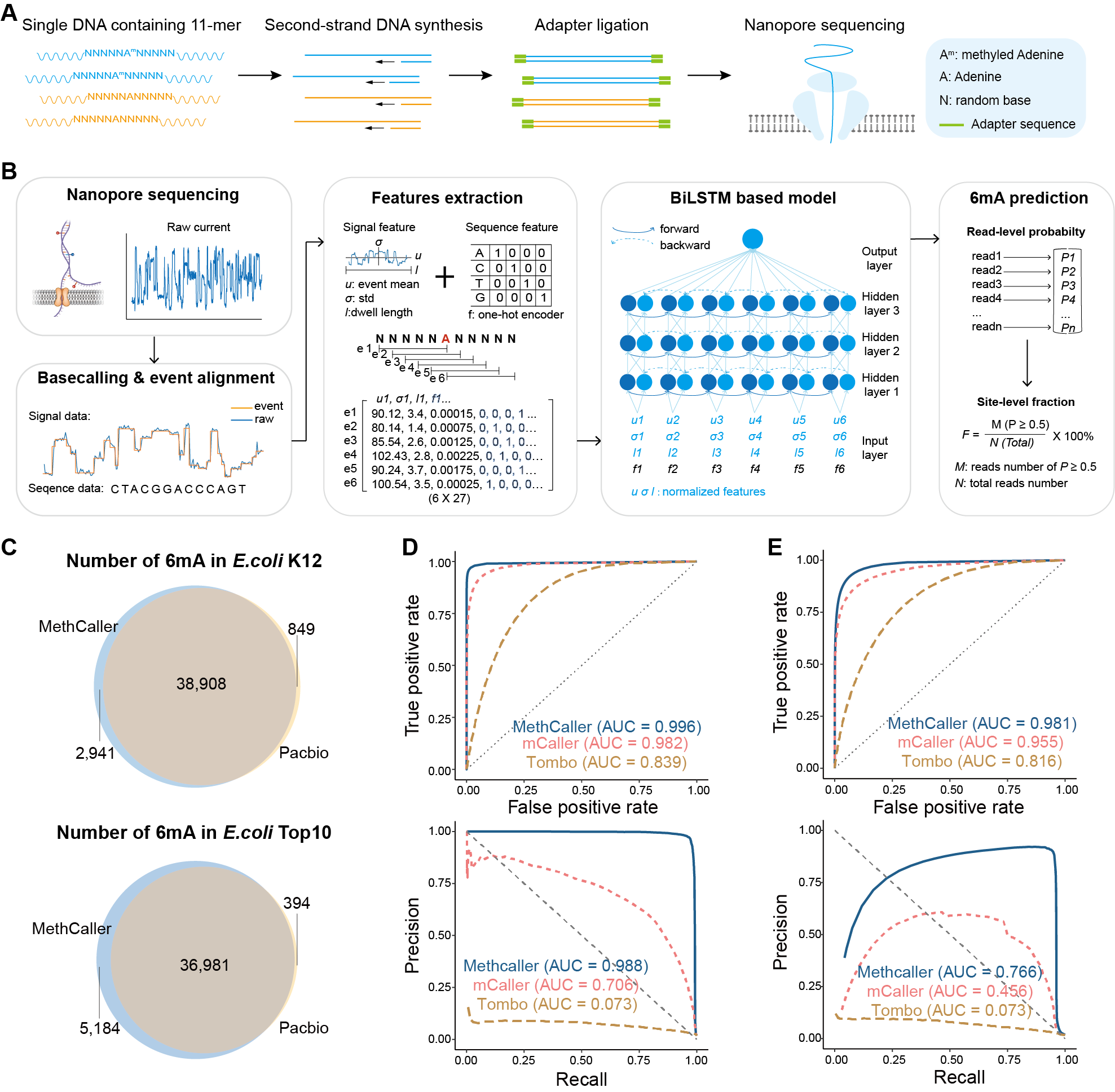
**

## Figure S1. Construction and evaluation of methCaller**.**

**A, Schematic diagram of the 11-mer library. B**, Schematic diagram of the methCaller model. Raw electrical signals were collected from ONT sequencing together with base-calling and event alignment. Signal features (including event mean, standard deviation and dwell time) and sequence features were then extracted separately and combined into our model, which contains a total of one input layer, three hidden layers and one output layer (see Methods for details). A probability of adenine methylation was given on each read, and finally the methylation fraction was calculated based on the probability of all the reads containing particular adenine-centered sequence in the genome. **C**, Benchmark of methCaller against Pacbio-derived 6mA methylation modification sites in *E. coli* K12 (top panel) and Top10 (bottom panel). **D** & **E**, Comparison of ROC curves (top) and PR curves (bottom) of methCaller, mCaller and Tombo based on the same *E. coli* k12 (**D**) and Top10 6mA data (**E**).


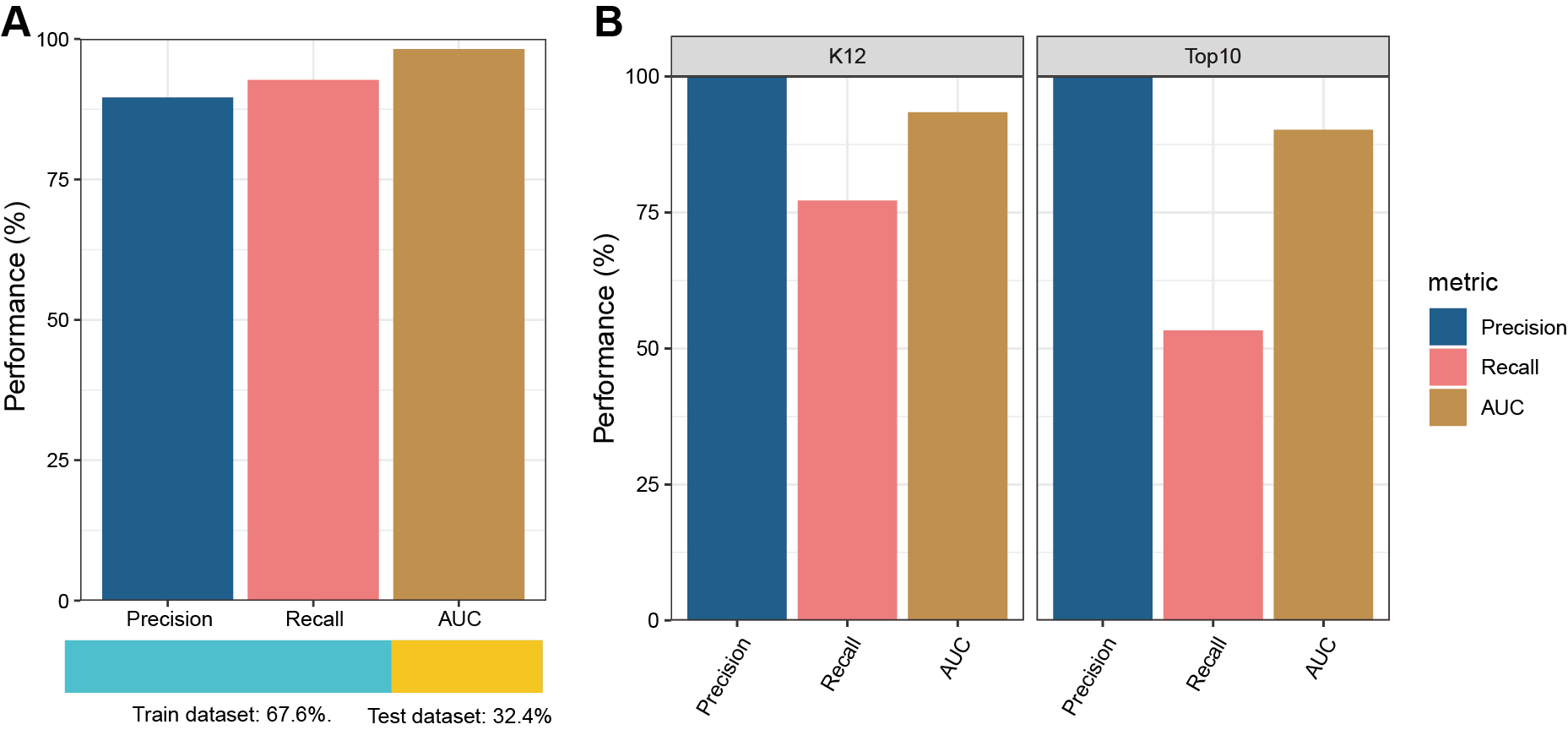


## **Figure S2. Evaluation of generalisation capabilities of methCaller.**

**A**, Barplot of precision, recall and AUC metrics obtained by methCaller using 67.6% of the data for training and 32.4% of the data for prediction. **B**, Faceted barplot of MethCaller prediction metrics for 11-mer motifs that are present in *E. coli* K12 and Top10 strains while not in the training set.

**
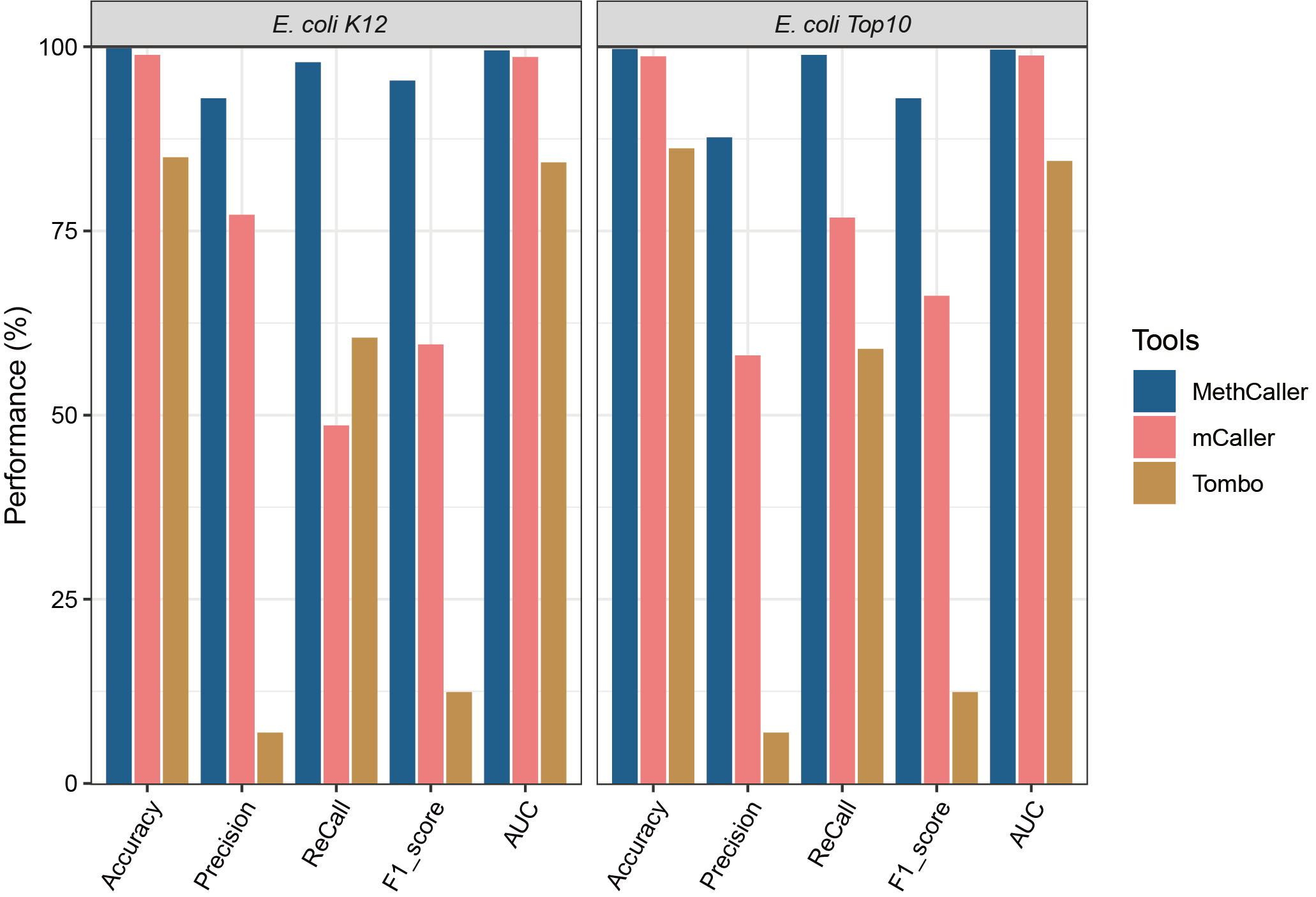
**

## **Figure S3. Performance comparison of methCaller against mCaller and Tombo.**

Faceted barplot of methCaller performance metrics compared to mCaller and Tombo on *E. coli* K12 and Top10 strains, respectively. All comparisons are based on 6mA methylation reference produced by Pacbio.

**
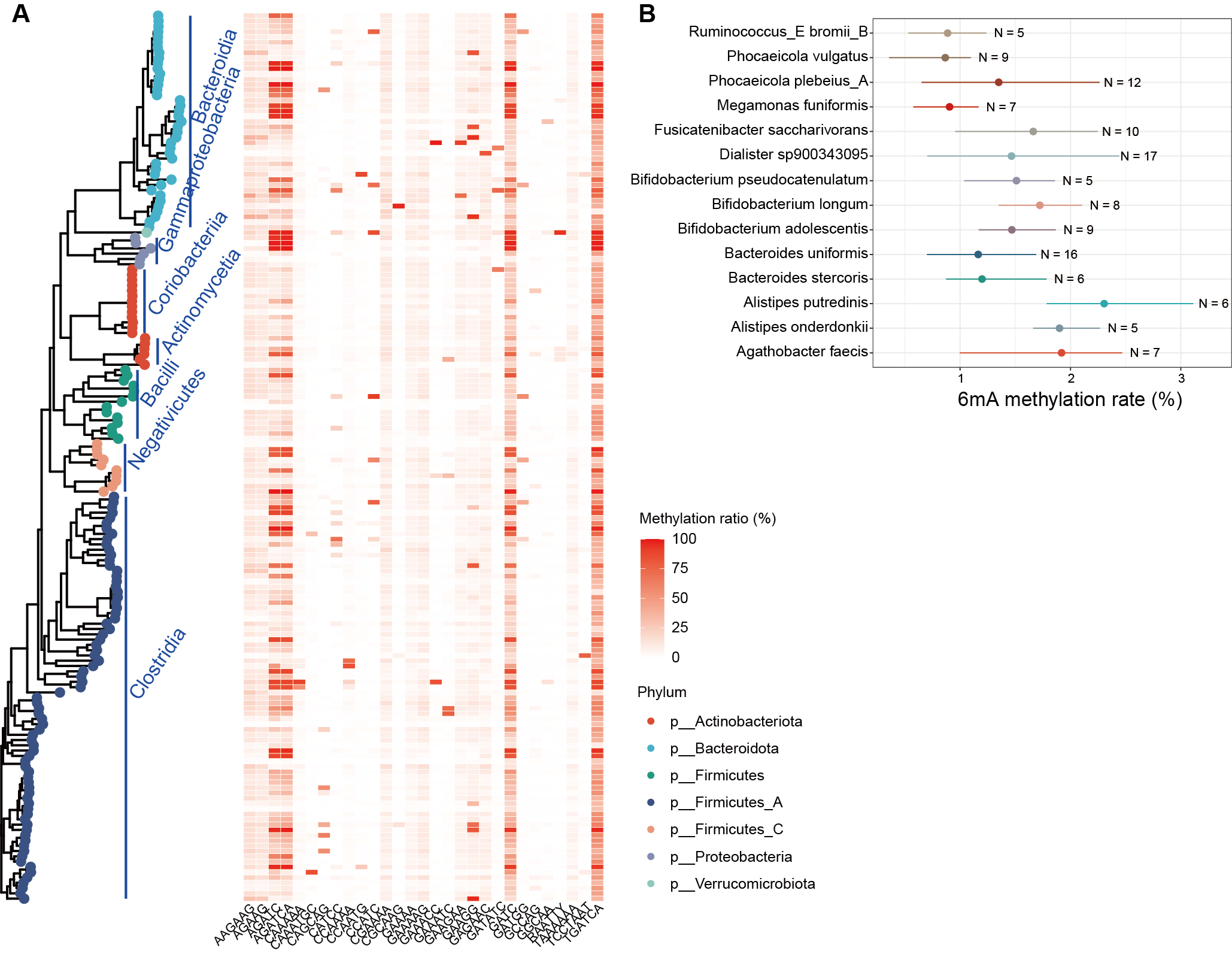
**

## **Figure S4. Landscape of 6mA methylation in human gut microbiome.**

**A**, Phylogenetic tree displaying the MAGs, together with heatmap of methylation ratios for detected motifs in each MAG. Node colors and text shows the phylogeny of MAGs at phylum level. The heatmap of the methylation ratio shows the highest levels detected for that MAG. **B**, Distribution of 6mA methylated ratios in intestinal bacterial genomes, calculated as the number of methylated adenines over the number of total adenines. The N number represents the number of times the bacteria appeared in the sample.

**
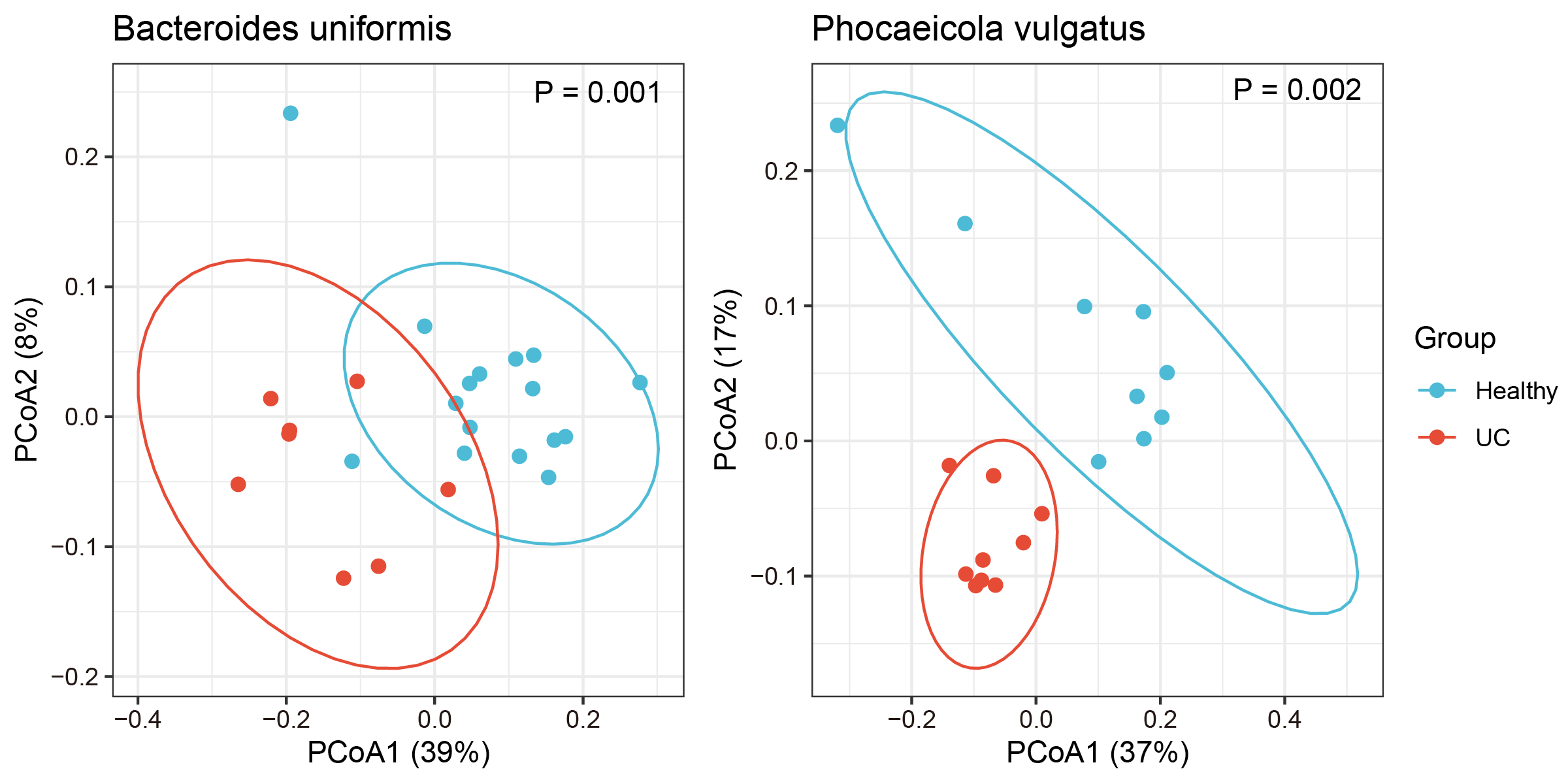
**

## **Figure S5. 6mA methylation of gut microbiota distinguishes healthy individuals from UC patients.**

PCoA plots of methylation levels of bacterial coding genes based on Bray-Curtis distance between healthy individuals and UC patients. Each point in plots above corresponds to one sample, ellipses represent 95% confidence intervals, and significance p-values are calculated by PER-MANOVA. The methylation levels of the bacterial coding genes involved in above were filtered by the ‘importance’ function in the ‘randomForest’ package using the metric ‘MeanDecreaseAccuracy’ > 1.

**
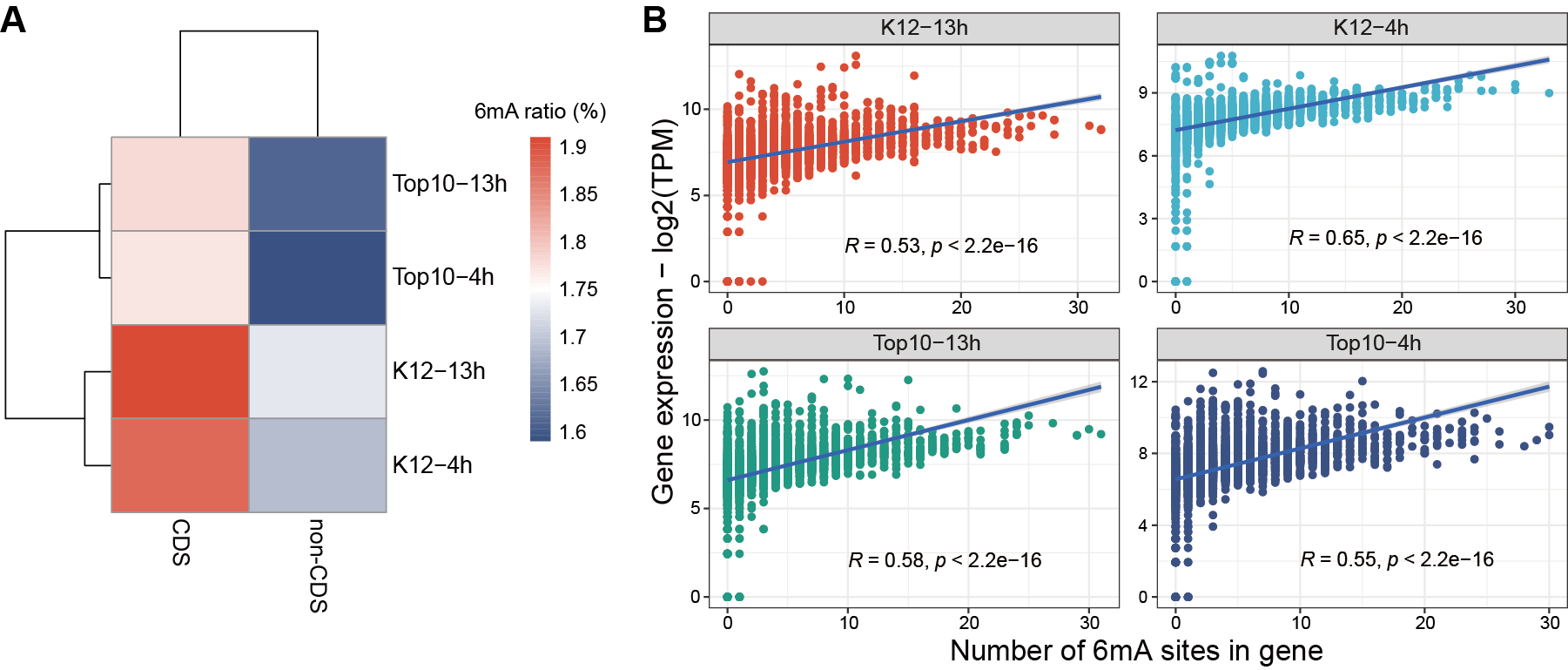
**

## **Figure S6. 6mA methylation in *E. coli* are positively correlated with gene expression.**

**A**, Heatmap of 6mA methylation levels in coding versus non-coding regions of the genome of E coli K12 and Top10 strains. The 4h and 13h represent two different growth stages of E. coli (logarithmic growth and plateau, respectively). 6mA ratio (%) is defined as the ratio of the number of methylated adenines to the total number of adenines. **b**, Scatterplot of correlation between 6mA methylation density and gene expression in genes encoded by E. coli K12 and Top10 strains at different growth stages.


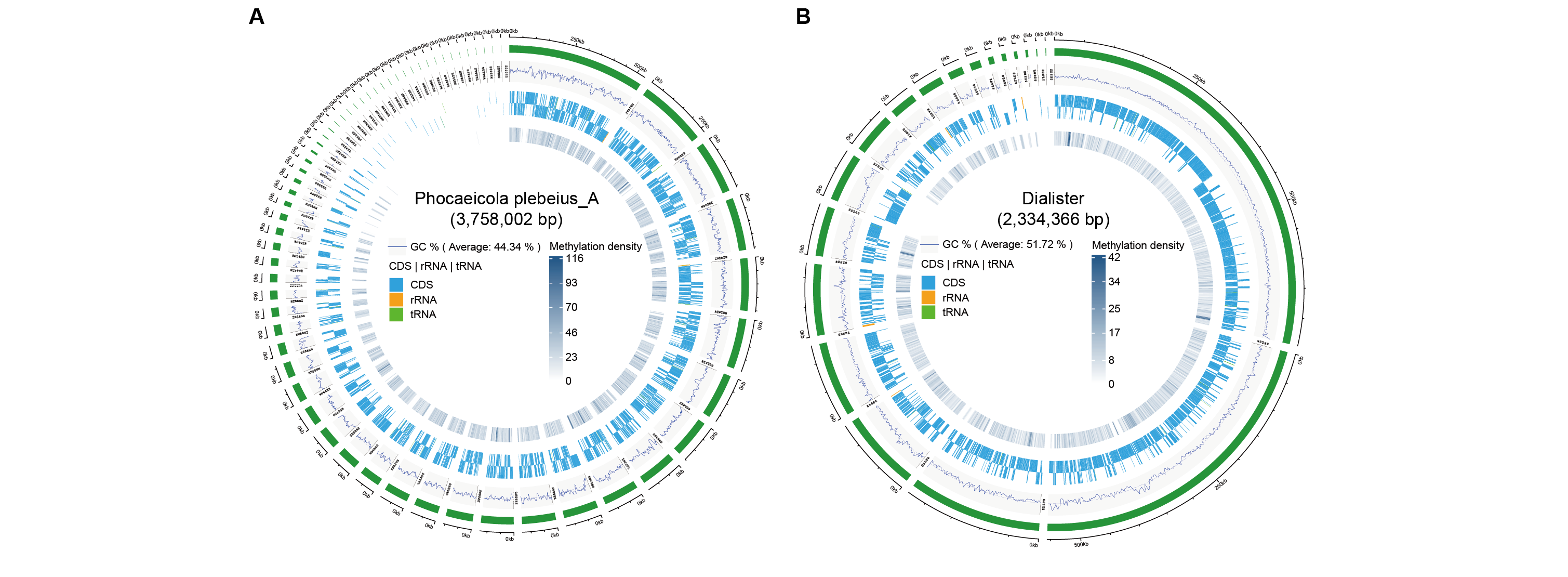


## **Figure S7. Distribution of 6mA methylation sites in intestinal bacterial genomes.**

**A**, **B**, Circle plots (a) and (b) show the distribution of 6mA methylation sites in the genomes of the human gut bacteria *Phocaeicola plebeius_A* and *Dialister*, respectively. Methylation density was obtained by counting the number of 6mA methylation sites per 2kb of genome.

**
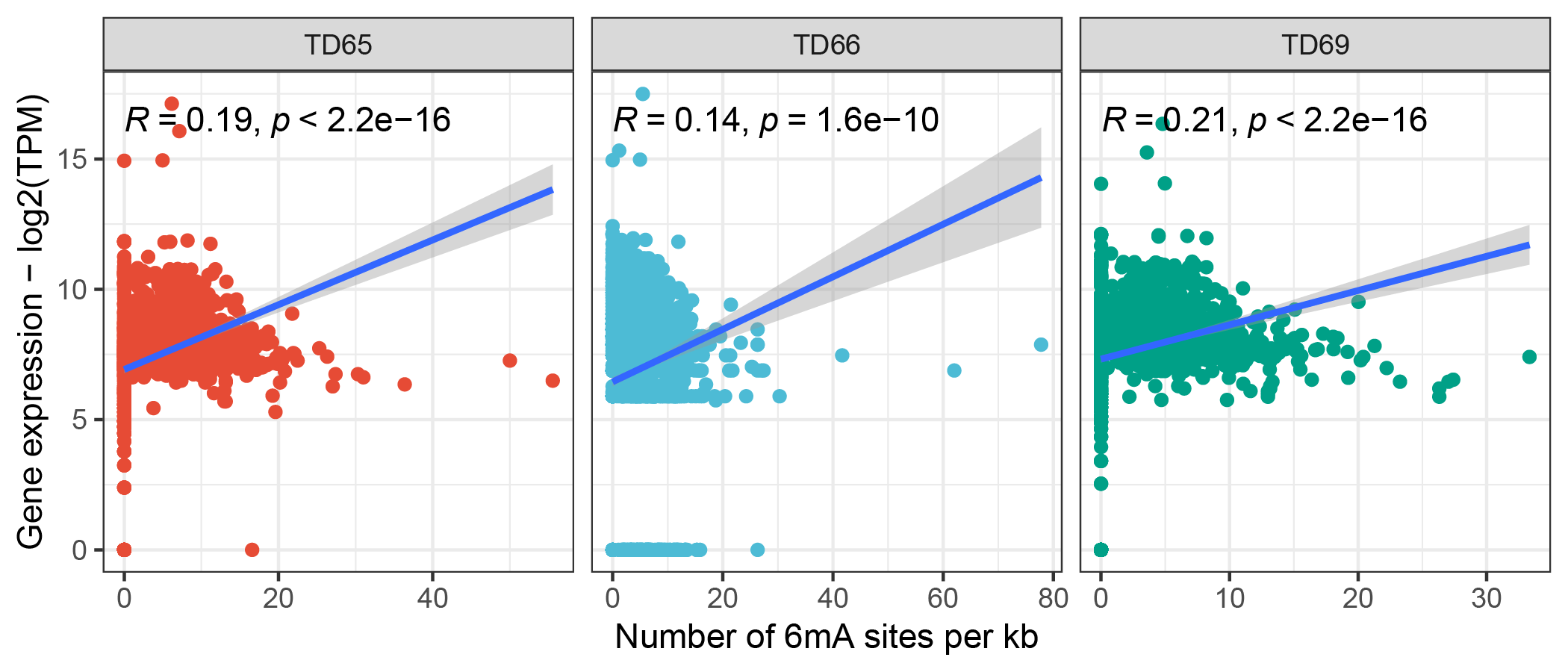
**

## **Figure S8. 6mA methylation in *Dialister* are positively correlated with gene expression**

**
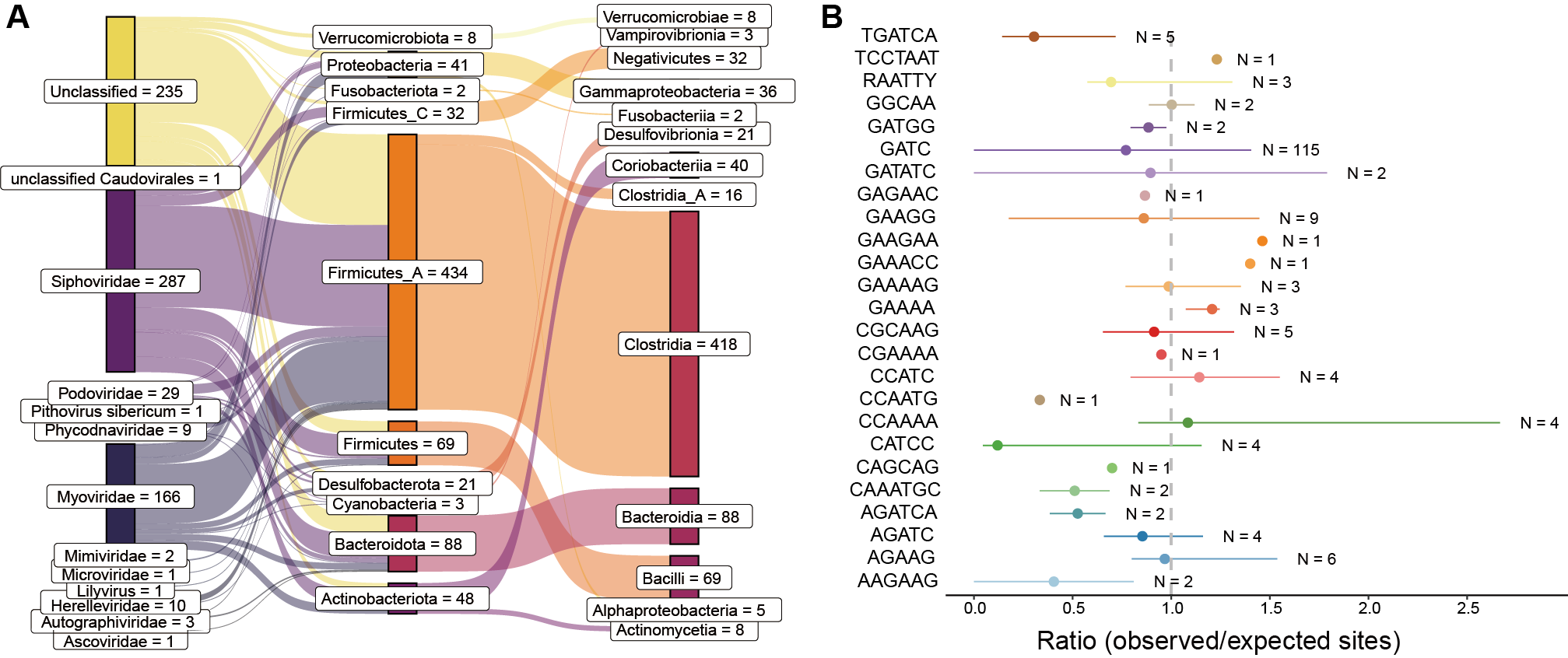
**

## **Figure S9. 6mA methylation of prophages in the human gut microbiome**

**A**, Sankey diagram of prophage-host interactions in the gut microbiome, columns from left to right indicate phage family level categorization, host phylum level and host class level categorization in that order, with numbers representing the number of genomes in each category. **B**, Ratio of observed over expected motif counts in prophages associated their host. The number of observed motifs is the number detected in the prophage genome, and the number of expected motifs is calculated from the ratio of their corresponding host motifs . The N number represents the number of prophages.

## **Supplementary Table 1. Denovo motifs identified by methCaller in human gut microbiome.**

| MAGs | Motifs | Curated motif | MethPos | Occurrence | p_value |
| --- | --- | --- | --- | --- | --- |
| CD100_100.0_0.0_bin.19.strict | HGATCDNSN | GATC | 2 | 27433 | 7.7e-447 |
| CD10_98.11_1.246_bin.32.strict | WHWTGATCAWN | TGATCA | 3 | 2285 | 4.7e-450 |
| CD10_98.11_1.246_bin.32.strict | GATC |  | 2 | 13967 | 1.2e-527 |
| CD12_98.43_1.342_bin.9.orig | GATC |  | 2 | 15203 | 3.5e-697 |
| CD12_99.25_0.0_bin.20.strict | GAAAA |  | 4 | 19199 | 2.4e-778 |
| CD12_99.98_1.497_bin.25.permissive | YGATC | GATC | 2 | 20719 | 2.2e-389 |
| CD13_99.37_0.0_bin.16.orig | GATC |  | 2 | 40881 | 1.4e-624 |
| CD13_99.90_0.0_bin.17.orig | GAAATCC | GAAATC | 4 | 5480 | 7.2e-1176 |
| CD15_91.85_0.210_bin.60.orig | GATC |  | 2 | 36293 | 2.9e-641 |
| CD15_95.96_0.946_bin.52.orig | GATC |  | 2 | 24433 | 3.6e-1036 |
| CD15_95.96_0.946_bin.52.orig | CAAATGC |  | 4 | 786 | 3.60E-275 |
| CD15_97.22_0.369_bin.2.orig | GATC |  | 2 | 24433 | 8.9e-615 |
| CD15_99.32_0.0_bin.62.strict | GATC |  | 2 | 26839 | 3.5e-754 |
| CD16_100.0_0.0_bin.30.orig | CCATC |  | 3 | 3376 | 4.9e-621 |
| CD16_100.0_0.0_bin.30.orig | GATGG |  | 2 | 3376 | 1.2e-341 |
| CD16_95.21_0.111_bin.3.orig | GATC |  | 2 | 6241 | 2.8e-450 |
| CD16_95.21_0.111_bin.3.orig | TAAAAAA |  | 6 | 3718 | 4.1e-735 |
| CD16_97.05_0.603_bin.66.orig | GATC |  | 2 | 49671 | 1.3e-681 |
| CD16_99.32_0.0_bin.14.orig | GATC |  | 2 | 12101 | 3.8e-628 |
| CD16_99.32_0.0_bin.14.orig | AGAAGAWRN | AGAAG | 4 | 43103 | 9.3e-333 |
| CD21_98.10_0.0_bin.28.orig | GATC |  | 2 | 55449 | 2.9e-748 |
| CD22_100.0_0.480_bin.30.strict | GATC |  | 2 | 35425 | 7.2e-1104 |
| CD25_98.98_0.788_bin.21.orig | GGCAA |  | 5 | 27069 | 2.0e-404 |
| CD25_98.98_0.788_bin.21.orig | YGATC | GATC | 2 | 12770 | 1.8e-488 |
| CD28_100.0_0.0_bin.35.orig | GATC |  | 2 | 21291 | 4.2e-885 |
| CD28_100.0_0.480_bin.18.strict | GATC |  | 2 | 30945 | 2.6e-1065 |
| CD2_94.09_0.632_bin.11.orig | GATCWB | GATC | 2 | 30827 | 3.3e-438 |
| CD29_98.38_0.0_bin.12.orig | AGAAGD | AGAAG | 4 | 16450 | 2.0e-521 |
| CD29_99.62_0.0_bin.32.orig | GATC |  | 2 | 33583 | 1.3e-1006 |
| CD31_90.01_1.641_bin.41.orig | GATC |  | 2 | 45095 | 4.0e-1074 |
| CD32_98.86_0.377_bin.42.orig | GATC |  | 2 | 16453 | 2.7e-767 |
| CD34_99.53_0.0_bin.46.orig | GATC |  | 2 | 25809 | 1.1e-1008 |
| CD35_93.28_0.0_bin.51.orig | GATC |  | 2 | 24561 | 7.3e-938 |
| CD35_98.07_0.384_bin.56.orig | CCAATG |  | 4 | 2442 | 1.2e-367 |
| CD35_99.19_0.0_bin.26.strict | GATC |  | 2 | 26071 | 3.8e-1151 |
| CD36_98.13_0.621_bin.28.orig | GATC |  | 2 | 30585 | 3.2e-749 |
| CD36_98.55_0.483_bin.18.orig | GAAGG |  | 3 | 4524 | 1.3e-629 |
| CD38_100.0_0.806_bin.38.orig | GATC |  | 2 | 19545 | 6.5e-990 |
| CD38_100.0_3.448_bin.55.strict | GATC |  | 2 | 32029 | 1.2e-1582 |
| CD39_96.49_0.641_bin.1.permissive | GATC |  | 2 | 15449 | 2.2e-1233 |
| CD39_98.29_0.0_bin.23.orig | GATC |  | 2 | 22685 | 9.1e-667 |
| CD40_85.16_9.523_bin.21.orig | VWGATC | GATC | 2 | 24819 | 8.0e-371 |
| CD41_78.12_0.894_bin.16.orig | TCCTAAT |  | 6 | 459 | 6.5e-403 |
| CD42_100.0_0.961_bin.12.strict | GATC |  | 2 | 2215 | 2.4e-919 |
| CD42_100.0_0.961_bin.12.strict | CGCAAG |  | 5 | 43983 | 8.2e-517 |
| CD45_94.50_0.0_bin.6.orig | GATC |  | 2 | 32589 | 3.9e-725 |
| CD48_98.65_0.0_bin.24.orig | GAAAAGSHCSNC | GAAAAG | 5 | 3293 | 2.8e-489 |
| CD48_98.65_0.0_bin.24.orig | GATC |  | 2 | 24699 | 2.7e-660 |
| CD49_100.0_0.0_bin.44.strict | GATC |  | 2 | 22885 | 7.3e-853 |
| CD51_98.63_0.0_bin.34.strict | CAGCAG |  | 5 | 6611 | 1.00E-236 |
| CD51_99.32_0.0_bin.30.orig | GATC |  | 2 | 44439 | 6.3e-786 |
| CD52_99.77_0.242_bin.31.strict | GATC |  | 2 | 24367 | 3.1e-906 |
| CD53_100.0_1.612_bin.48.permissive | GATC |  | 2 | 20193 | 2.3e-1560 |
| CD55_100.0_0.806_bin.25.orig | GATC |  | 2 | 20011 | 3.2e-996 |
| CD59_99.32_1.342_bin.19.strict | DNMAGAAGWW | AGAAG | 4 | 8116 | 1.7e-495 |
| CD60_100.0_0.0_bin.6.orig | GATC |  | 2 | 40119 | 3.8e-824 |
| CD60_99.41_0.0_bin.7.permissive | GATC |  | 2 | 41909 | 5.0e-689 |
| CD65_99.40_0.299_bin.18.orig | CCATC |  | 3 | 2427 | 4.4e-349 |
| CD66_95.56_0.0_bin.2.orig | GATC |  | 2 | 30295 | 4.3e-711 |
| CD67_99.53_0.311_bin.16.strict | DGATC | GATC | 2 | 5899 | 1.7e-938 |
| CD69_97.98_0.0_bin.9.orig | GATC |  | 2 | 29019 | 2.9e-926 |
| CD72_91.94_0.671_bin.17.orig | GATC |  | 2 | 19923 | 2.7e-728 |
| CD74_94.30_0.0_bin.4.orig | GATC |  | 2 | 28431 | 2.4e-778 |
| CD77_99.47_0.0_bin.34.orig | AAGAAG |  | 5 | 3108 | 2.5e-377 |
| CD78_98.65_0.0_bin.39.orig | GATC |  | 2 | 17282 | 1.4e-744 |
| CD78_98.65_0.0_bin.39.orig | GAAAA |  | 4 | 29039 | 3.3e-602 |
| CD79_100.0_0.0_bin.31.strict | GATC |  | 2 | 26545 | 1.3e-671 |
| CD80_91.05_0.0_bin.8.orig | GATC |  | 2 | 26841 | 1.2e-1074 |
| CD82_97.98_1.342_bin.43.orig | GATC |  | 2 | 40151 | 3.2e-944 |
| CD82_99.51_0.0_bin.28.permissive | GATC |  | 2 | 4518 | 3.4e-642 |
| CD82_99.51_0.0_bin.28.permissive | CGAAAA |  | 5 | 38089 | 9.8e-400 |
| CD84_100.0_0.806_bin.12.orig | GATC |  | 2 | 19821 | 1.2e-1021 |
| CD84_97.32_0.272_bin.2.orig | AGATC |  | 3 | 6812 | 5.1e-407 |
| CD88_100.0_0.0_bin.10.strict | CAAAAA |  | 6 | 6284 | 1.4e-665 |
| CD88_100.0_0.806_bin.31.orig | GATC |  | 2 | 20081 | 7.5e-687 |
| CD88_99.05_0.0_bin.7.orig | WWGATC | GATC | 2 | 14627 | 8.0e-425 |
| CD8_95.56_0.0_bin.17.orig | GATC |  | 2 | 55333 | 2.1e-735 |
| CD89_99.25_0.231_bin.19.orig | GATATC |  | 2 | 1723 | 1.40E-204 |
| CD91_100.0_0.0_bin.55.orig | TGATCA |  | 3 | 4485 | 8.1e-828 |
| CD91_87.40_0.949_bin.45.orig | TGATCANNNNNNH | TGATCA | 3 | 4083 | 5.2e-734 |
| CD92_100.0_0.037_bin.46.strict | GATC |  | 2 | 59863 | 2.0e-1526 |
| CD92_90.73_0.957_bin.50.orig | GAAGG |  | 3 | 9414 | 1.7e-488 |
| CD92_97.95_0.0_bin.41.permissive | RAATTY |  | 3 | 8949 | 9.2e-536 |
| CD92_99.19_1.075_bin.20.orig | GATC |  | 2 | 7445 | 4.7e-673 |
| CD92_99.19_1.075_bin.20.orig | CCAAAA |  | 6 | 677 | 1.0e-351 |
| CD93_87.91_0.0_bin.15.permissive | GATC |  | 2 | 9809 | 3.4e-1382 |
| CD93_91.88_1.342_bin.12.orig | GATC |  | 2 | 30305 | 1.7e-1390 |
| CD93_97.09_0.0_bin.56.strict | GATC |  | 2 | 11601 | 9.0e-1246 |
| CD93_98.38_0.0_bin.53.orig | CCAAAA |  | 6 | 1236 | 2.8e-478 |
| CD94_100.0_0.632_bin.42.orig | GATC |  | 2 | 34007 | 2.2e-1116 |
| CD95_89.48_0.561_bin.14.orig | GATC |  | 2 | 2967 | 4.8e-687 |
| CD95_98.91_0.603_bin.11.orig | GATC |  | 2 | 52689 | 7.8e-609 |
| CD96_99.25_0.972_bin.57.orig | GAAGAA |  | 6 | 1488 | 1.8e-796 |
| CD96_99.25_0.972_bin.57.orig | GAAACC |  | 4 | 4308 | 6.5e-424 |
| CD98_93.28_0.0_bin.23.orig | GATC |  | 2 | 26605 | 1.9e-915 |
| CD99_99.41_0.0_bin.6.orig | GATC |  | 2 | 37437 | 1.7e-725 |
| TD100_99.18_0.0_bin.12.strict | GATC |  | 2 | 27327 | 2.4e-990 |
| TD10_99.53_0.123_bin.68.orig | GAAGG |  | 3 | 11995 | 7.0e-861 |
| TD1_100.0_0.0_bin.16.orig | GATC |  | 2 | 24623 | 2.0e-981 |
| TD15_95.97_0.671_bin.18.orig | GATC |  | 2 | 27023 | 1.9e-930 |
| TD16_98.65_0.671_bin.19.orig | GATC |  | 2 | 48149 | 2.0e-823 |
| TD17_86.37_0.155_bin.7.strict | GATC |  | 2 | 32175 | 1.5e-1243 |
| TD18_100.0_0.0_bin.20.orig | AGATCA |  | 3 | 1990 | 8.2e-442 |
| TD23_99.36_0.0_bin.11.orig | GATC |  | 2 | 37869 | 2.6e-739 |
| TD27_99.96_0.037_bin.4.orig | GATC |  | 2 | 38301 | 1.9e-682 |
| TD28_99.42_0.128_bin.8.orig | HGATCNN | GATC | 2 | 60427 | 1.3e-517 |
| TD2_95.84_1.342_bin.33.orig | GATC |  | 2 | 26131 | 1.4e-993 |
| TD2_98.65_0.0_bin.43.orig | GATC |  | 2 | 30685 | 3.6e-584 |
| TD3_100.0_0.806_bin.51.orig | GATC |  | 2 | 20121 | 1.9e-900 |
| TD33_91.81_0.0_bin.31.orig | GATC |  | 2 | 36067 | 1.7e-678 |
| TD33_96.08_0.0_bin.10.orig | GATC |  | 2 | 22185 | 1.4e-828 |
| TD33_98.94_0.0_bin.20.orig | NMNGATCNKNNNNN | GATC | 2 | 36731 | 1.8e-353 |
| TD37_99.62_0.0_bin.22.orig | GATC |  | 2 | 28561 | 8.6e-1129 |
| TD38_100.0_0.0_bin.39.orig | GATC |  | 2 | 46097 | 8.4e-722 |
| TD3_99.41_0.0_bin.37.orig | GATCWB | GATC | 2 | 36359 | 7.2e-475 |
| TD44_99.32_0.0_bin.18.orig | CATCC |  | 2 | 7537 | 5.1e-548 |
| TD47_100.0_0.806_bin.51.strict | GATC |  | 2 | 19931 | 1.5e-933 |
| TD47_100.0_0.806_bin.51.strict | GCCAG |  | 4 | 6528 | 2.2e-406 |
| TD50_98.65_0.0_bin.35.orig | GATC |  | 2 | 42401 | 8.4e-760 |
| TD51_99.46_0.0_bin.15.orig | YGATC | GATC | 2 | 43103 | 1.1e-436 |
| TD52_99.27_0.480_bin.11.orig | GATC |  | 2 | 36075 | 1.6e-648 |
| TD5_98.24_0.0_bin.24.orig | GATC |  | 2 | 37555 | 4.6e-756 |
| TD59_99.36_0.316_bin.38.orig | GATC |  | 2 | 53773 | 9.2e-710 |
| TD60_100.0_0.806_bin.10.orig | GATC |  | 2 | 19923 | 1.3e-1050 |
| TD64_100.0_0.0_bin.28.orig | GATCD | GATC | 2 | 23191 | 1.7e-493 |
| TD65_97.98_0.0_bin.21.orig | GATC |  | 2 | 21749 | 1.1e-636 |
| TD69_99.32_0.0_bin.20.strict | GATCHNNNNNNN | GATC | 2 | 28159 | 5.6e-685 |
| TD74_100.0_0.806_bin.13.orig | GATC |  | 2 | 20333 | 1.5e-829 |
| TD79_97.82_1.187_bin.1.strict | GATC |  | 2 | 48291 | 1.4e-684 |
| TD84_98.73_0.632_bin.54.orig | GATC |  | 2 | 50959 | 5.3e-902 |
| TD87_97.18_0.123_bin.2.orig | GAGAAC |  | 4 | 1075 | 6.0e-432 |
| TD87_98.49_0.0_bin.17.orig | GAAGG |  | 3 | 8131 | 1.9e-800 |
| TD89_94.56_1.570_bin.3.strict | GATC |  | 2 | 44209 | 4.1e-793 |
| TD90_100.0_0.077_bin.15.orig | AGATC |  | 3 | 6933 | 4.3e-938 |
| TD90_100.0_0.077_bin.15.orig | AGAAG |  | 4 | 11666 | 3.7e-415 |
| TD90_95.96_0.0_bin.18.orig | AGAAGDW | AGAAG | 4 | 12860 | 4.2e-477 |
| TD90_99.37_0.294_bin.48.orig | GATC |  | 2 | 19107 | 4.0e-982 |
| TD91_98.55_0.0_bin.37.orig | GATC |  | 2 | 31603 | 1.1e-919 |
| TD92_100.0_0.0_bin.27.orig | GATC |  | 2 | 6887 | 3.4e-910 |
| TD94_100.0_0.0_bin.26.orig | YGATC | GATC | 2 | 26769 | 4.0e-431 |
| TD97_100.0_0.806_bin.15.orig | GATC |  | 2 | 20095 | 2.9e-870 |
| TD9_89.59_0.0_bin.60.orig | GATC |  | 2 | 17321 | 2.4e-710 |
| TD9_93.96_1.690_bin.32.orig | GATC |  | 2 | 48099 | 2.2e-1154 |

**REFERENCE**

1 Kahramanoglou, C. *et al.* Genomics of DNA cytosine methylation in Escherichia coli reveals its role in stationary phase transcription. *Nat Commun* **3**, 886, doi:10.1038/ncomms1878 (2012).

2 Zweiger, G., Marczynski, G. & Shapiro, L. A Caulobacter DNA methyltransferase that functions only in the predivisional cell. *Journal of molecular biology* **235**, 472-485, doi:10.1006/jmbi.1994.1007 (1994).

3 Nelson, M., Raschke, E. & McClelland, M. Effect of site-specific methylation on restriction endonucleases and DNA modification methyltransferases. *Nucleic Acids Res* **21**, 3139-3154, doi:10.1093/nar/21.13.3139 (1993).

4 Fang, G. *et al.* Genome-wide mapping of methylated adenine residues in pathogenic Escherichia coli using single-molecule real-time sequencing. *Nature biotechnology* **30**, 1232-1239, doi:10.1038/nbt.2432 (2012).

5 Flusberg, B. A. *et al.* Direct detection of DNA methylation during single-molecule, real-time sequencing. *Nature methods* **7**, 461-465, doi:10.1038/nmeth.1459 (2010).

6 Schadt, E. E. *et al.* Modeling kinetic rate variation in third generation DNA sequencing data to detect putative modifications to DNA bases. *Genome research* **23**, 129-141, doi:10.1101/gr.136739.111 (2013).

7 Rand, A. C. *et al.* Mapping DNA methylation with high-throughput nanopore sequencing. *Nature methods* **14**, 411-413, doi:10.1038/nmeth.4189 (2017).

8 McIntyre, A. B. R. *et al.* Single-molecule sequencing detection of N6-methyladenine in microbial reference materials. *Nat Commun* **10**, 579, doi:10.1038/s41467-019-08289-9 (2019).

9 Ni, P. *et al.* DeepSignal: detecting DNA methylation state from Nanopore sequencing reads using deep-learning. *Bioinformatics (Oxford, England)* **35**, 4586-4595, doi:10.1093/bioinformatics/btz276 (2019).

10 Liu, Q. *et al.* Detection of DNA base modifications by deep recurrent neural network on Oxford Nanopore sequencing data. *Nat Commun* **10**, 2449, doi:10.1038/s41467-019-10168-2 (2019).

11 Stoiber, M. *et al.* De novo Identification of DNA Modifications Enabled by Genome-Guided Nanopore Signal Processing. *bioRxiv*, 094672, doi:10.1101/094672 (2017).

12 Tourancheau, A., Mead, E. A., Zhang, X.-S. & Fang, G. Discovering multiple types of DNA methylation from bacteria and microbiome using nanopore sequencing. *Nat. Methods* **18**, 491-498, doi:10.1038/s41592-021-01109-3 (2021).

13 Charalampous, T. *et al.* Nanopore metagenomics enables rapid clinical diagnosis of bacterial lower respiratory infection. *Nat. Biotechnol* **37**, 783-792, doi:10.1038/s41587-019-0156-5 (2019).

14 Wang, Y., Zhao, Y., Bollas, A., Wang, Y. & Au, K. F. Nanopore sequencing technology, bioinformatics and applications. *Nat. Biotechnol* **39**, 1348-1365, doi:10.1038/s41587-021-01108-x (2021).

15 Chen, L. *et al.* Short- and long-read metagenomics expand individualized structural variations in gut microbiomes. *Nat Commun* **13**, 3175, doi:10.1038/s41467-022-30857-9 (2022).

16 Li, H. Minimap2: pairwise alignment for nucleotide sequences. *Bioinformatics (Oxford, England)* **34**, 3094-3100, doi:10.1093/bioinformatics/bty191 %J Bioinformatics (2018).

17 Loman, N. J., Quick, J. & Simpson, J. T. A complete bacterial genome assembled de novo using only nanopore sequencing data. *Nat. Methods* **12**, 733-735, doi:10.1038/nmeth.3444 (2015).

18 Shen, W., Le, S., Li, Y. & Hu, F. SeqKit: A Cross-Platform and Ultrafast Toolkit for FASTA/Q File Manipulation. *PLOS ONE* **11**, e0163962, doi:10.1371/journal.pone.0163962 (2016).

19 Quinlan, A. R. & Hall, I. M. BEDTools: a flexible suite of utilities for comparing genomic features. *Bioinformatics (Oxford, England)* **26**, 841-842, doi:10.1093/bioinformatics/btq033 %J Bioinformatics (2010).

20 Seemann, T. Prokka: rapid prokaryotic genome annotation. *Bioinformatics (Oxford, England)* **30**, 2068-2069, doi:10.1093/bioinformatics/btu153 (2014).

21 Bailey, T. L., Johnson, J., Grant, C. E. & Noble, W. S. The MEME Suite. *Nucleic Acids Res* **43**, W39-49, doi:10.1093/nar/gkv416 (2015).

22 Kieft, K., Zhou, Z. & Anantharaman, K. VIBRANT: automated recovery, annotation and curation of microbial viruses, and evaluation of viral community function from genomic sequences. *Microbiome* **8**, 90, doi:10.1186/s40168-020-00867-0 (2020).

23 Huson, D. H., Auch, A. F., Qi, J. & Schuster, S. C. MEGAN analysis of metagenomic data. *Genome research* **17**, 377-386, doi:10.1101/gr.5969107 (2007).
